# Impaired regulation of heart rate and sinoatrial node function by the parasympathetic nervous system in type 2 diabetic mice

**DOI:** 10.1038/s41598-021-91937-2

**Published:** 2021-06-14

**Authors:** Yingjie Liu, Hailey J. Jansen, Pooja S. Krishnaswamy, Oleg Bogachev, Robert A. Rose

**Affiliations:** 1 Department of Cardiac Sciences, Libin Cardiovascular Institute, Cumming School of Medicine, University of Calgary, GAC66, Health Research Innovation Centre, 3280 Hospital Drive N.W., Calgary, AB T2N 4Z6 Canada; 2 Department of Physiology and Pharmacology, Libin Cardiovascular Institute, Cumming School of Medicine, University of Calgary, GAC66, Health Research Innovation Centre, 3280 Hospital Drive N.W., Calgary, AB T2N 4Z6 Canada; 3 Department of Physiology and Biophysics, Dalhousie University, Halifax, NS Canada

**Keywords:** Arrhythmias, Cardiovascular diseases

## Abstract

Heart rate (HR) and sinoatrial node (SAN) function are modulated by the autonomic nervous system. HR regulation by the parasympathetic nervous system (PNS) is impaired in diabetes mellitus (DM), which is denoted cardiovascular autonomic neuropathy. Whether blunted PNS effects on HR in type 2 DM are related to impaired responsiveness of the SAN to PNS agonists is unknown. This was investigated in type 2 diabetic db/db mice in vivo and in isolated SAN myocytes. The PNS agonist carbachol (CCh) had a smaller inhibitory effect on HR, while HR recovery time after CCh removal was accelerated in db/db mice. In isolated SAN myocytes CCh reduced spontaneous action potential firing frequency but this effect was reduced in db/db mice due to blunted effects on diastolic depolarization slope and maximum diastolic potential. Impaired effects of CCh occurred due to enhanced desensitization of the acetylcholine-activated K^+^ current (I_KACh_) and faster I_KACh_ deactivation. I_KACh_ alterations were reversed by inhibition of regulator of G-protein signaling 4 (RGS4) and by the phospholipid PIP_3_. SAN expression of RGS4 was increased in db/db mice. Impaired PNS regulation of HR in db/db mice occurs due to reduced responsiveness of SAN myocytes to PNS agonists in association with enhanced RGS4 activity.

## Introduction

Heart rate (HR), which is a critical determinant of cardiac performance, is determined by the intrinsic properties of the sinoatrial node (SAN) and is modulated by the autonomic nervous system (ANS)^1,2^. The sympathetic nervous system (SNS) increases HR by enhancing SAN activity while the parasympathetic nervous system (PNS) reduces HR via the actions of acetylcholine on muscarinic receptors (M_2_R) in the SAN^2^.

In diabetes mellitus (DM), cardiovascular complications are highly prevalent, leading to death and morbidity in DM patients. HR regulation by the ANS is known to be impaired in DM patients, which has been attributed to a condition denoted cardiovascular autonomic neuropathy (CAN)^3,4^. CAN, which can be associated with damage to the nerves that innervate the heart, can affect up to 90% of DM patients and increases mortality by two- to five-fold compared to DM patients without CAN^3,5^. Although nerve damage may be involved in the impairments in HR regulation by the PNS, it is also possible that these impairments may occur due to alterations in PNS signaling within the SAN.

SAN myocytes generate spontaneous action potentials (APs), which set the intrinsic HR, in association with a diastolic depolarization (DD) that occurs between successive APs^2^. The DD is generated by a number of ionic currents including the hyperpolarization-activated current (I_f_), which is generated by hyperpolarization-activated cyclic nucleotide gated (HCN) channels, and a rapidly-activating delayed rectifier K^+^ currents (I_Kr_), which is generated by *ether-a-go-go* (ERG) channels^1,6^. The PNS reduces HR via the activation of inhibitory G proteins (G_i_) associated with M_2_Rs. Key mediators of this reduction in HR are the acetylcholine-activated K^+^ current (I_KACh_; generated by K_ir_3.1 and K_ir_3.4 channels) and I_f_^1,7^. Specifically, activation of I_KACh_ by the βγ subunit of the G_i_ protein results in hyperpolarization of the maximum diastolic potential (MDP) in SAN myocytes while inhibition of I_f_ downstream of G_αi_ and a reduction in cyclic AMP (cAMP) reduces the slope of the DD in SAN myocytes. Both effects contribute importantly to a slowing of spontaneous AP firing in SAN myocytes.

Previous studies have shown that regulation of HR by the PNS is impaired in type 1 DM (T1DM) and that this occurs in association with altered responsiveness to PNS agonists in the SAN^8,9^. Type 2 DM (T2DM) accounts for up to 90% of all DM patients and is also associated with impaired PNS activity^4,10^. Importantly, blunted PNS activity and CAN present earlier in T2DM patients compared to T1DM^4^; however, the basis for this is unknown making studies of dysregulation of HR by the PNS specifically in T2DM essential. Accordingly, the purpose of this study was to investigate the regulation of HR and SAN function by the PNS using db/db mice, a model of obesity and T2DM^11^.

## Methods

An expanded methods section is available in the Supplementary Information.

### Animals

This study used male and female littermate wildtype and db/db (strain: C57BL/gj-*Lepr*^*db*^) mice between 16 and 20 weeks of age. The db/db mice contain a mutation in the leptin receptor (*Lepr*) gene leading to hyperphagia^11^. These db/db mice exhibit the expected features of T2DM including obesity and hyperglycemia, as we^12^ and others^13,14^ have shown. All experimental procedures were approved by the University of Calgary Animal Care and Use Committee or the Dalhousie University Committee for Laboratory Animals and were in accordance with the guidelines of the Canadian Council on Animal Care and the ARRIVE guidelines.

### Intracardiac electrophysiology and electrocardiogram recording

HR was measured from lead II surface electrocardiograms (ECGs) and corrected SAN recovery time (cSNRT) was measured using an octapolar electrophysiology catheter in anesthetized mice as described previously^8,15,16^ and in the Supplementary Information.

### Patch-clamping of isolated sinoatrial node myocytes

Isolated SAN myocytes were used to record spontaneous APs and ionic currents, including I_f_ and I_Kr_, using the whole cell patch-clamp technique in current clamp or voltage clamp mode, respectively. The protocols and solutions for these experiments are described in the Supplementary Information.

### Quantitative polymerase chain reaction and Western blotting

Quantitative gene expression was measured in isolated SAN tissue as previously described^16,17^. Western blotting was performed using isolated SAN tissue as described previously^16^. The experimental protocols for these techniques are described in the Supplementary Information.

### Statistical analysis

All data are presented as means ± SEM. Data were analyzed using two-way ANOVA with the Holm–Sidak posthoc test or Student’s *t*-test as indicated in each figure legend. *P* < 0.05 was considered significant.

## Results

### Effects of carbachol on heart rate and sinoatrial node function in db/db diabetic mice

**Figure 1:**
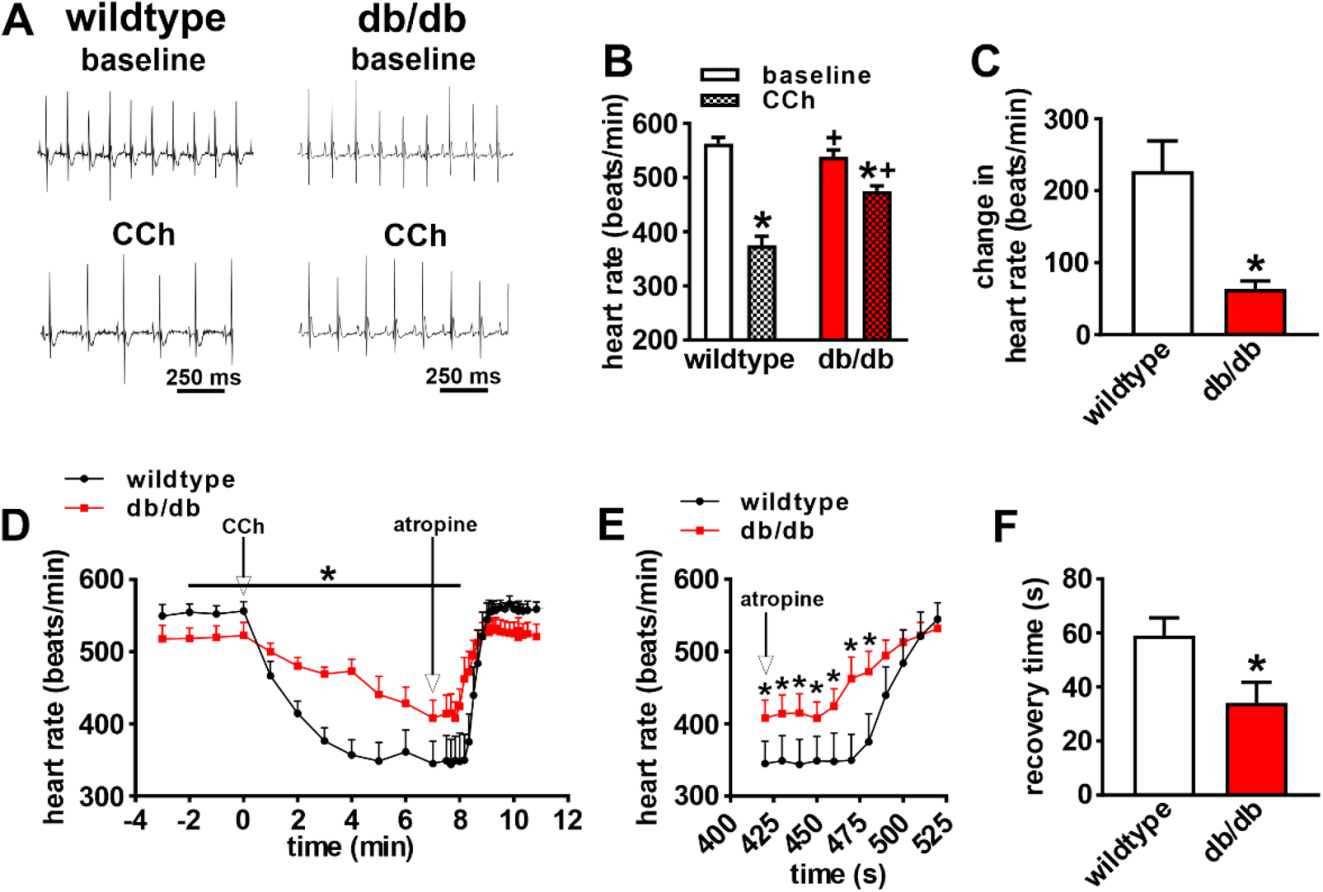
Effects of carbachol on heart rate in db/db mice in vivo. (**A**) Representative surface ECGs in baseline conditions and after application of CCh (0.1 mg/kg) in anesthetized wildtype and db/db mice. (**B**) Summary of heart rate at baseline and after CCh in wildtype (*n* = 9) and db/db (*n* = 13) mice. *P < 0.05 vs baseline; ^+^*P* < 0.05 vs. wildtype by two-way ANOVA with Holm–Sidak posthoc test. (**C**) Change in heart rate after CCh application in wildtype and db/db mice. *P < 0.05 vs. wildtype by Student’s t-test. (**D**) Time course of changes in heart rate after application of CCh and atropine (10 mg/kg) in wildtype (*n* = 5) and db/db (*n* = 6) mice. (**E**) Magnified region of data from panel D illustrating the changes in heart rate upon application of atropine in wildtype and db/db mice. For panels (**D**) and (**E**) *P < 0.05 vs. wildtype by two-way repeated measures ANOVA with Holm–Sidak posthoc test. (**F**) Recovery time for heart rate after application of atropine. *P < 0.05 vs. wildtype by Student’s *t*-test.

**Figure 2:**
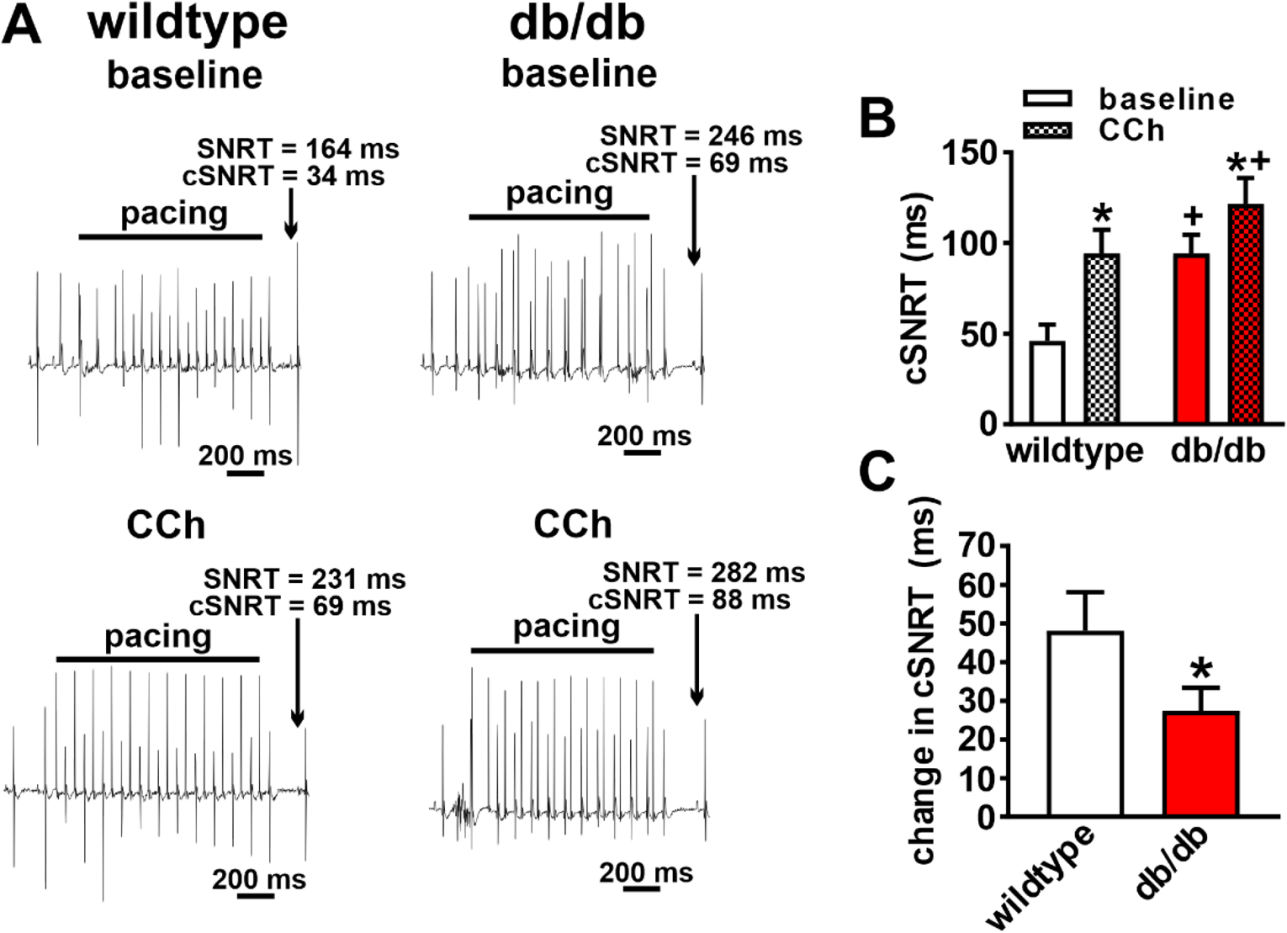
Effects of carbachol on sinoatrial node recovery time in db/db mice in vivo. (**A**) Representative recordings illustrating assessment of corrected sinoatrial node recovery time (cSNRT) at baseline and after CCh (0.1 mg/kg) in wildtype and db/db mice. (**B**) Summary of cSNRT at baseline and after CCh in wildtype (*n* = 7) and db/db (*n* = 11) mice. *P < 0.05 vs baseline; ^+^*P* < 0.05 vs. wildtype by two-way ANOVA with Holm–Sidak posthoc test. (**C**) Change in cSNRT after CCh application in wildtype and db/db mice. *P < 0.05 vs. wildtype by Student’s *t*-test.

ECG recordings in anesthetized mice (Fig. 1A) demonstrate that HR was lower in db/db mice at baseline and that the ability of the PNS agonist carbachol (CCh; 0.1 mg/kg intraperitoneal injection) to reduce HR was reduced compared to wildtype mice (Fig. 1B,C). The kinetics of the effects of CCh on HR, and the return of HR to baseline after application of the muscarinic (M_2_) receptor blocker atropine (10 mg/kg, intraperitoneal injection), are presented in Fig. 1D–F. These data further demonstrate that the ability of CCh to reduce HR is impaired in db/db mice and that the return to baseline HR after application of atropine occurs faster in db/db mice (Fig. 1D–F). Impaired HR regulation by CCh in db/db mice was also studied by directly assessing SAN function, as determined by corrected SAN recovery time (cSNRT), in vivo (Fig. 2A). cSNRT was longer at baseline in db/db mice. Application of CCh (0.1 mg/kg) prolonged cSNRT; however, this response was reduced in db/db mice (Fig. 2B,C). These data indicate that the effects of CCh on HR are reduced in db/db mice and that this is associated with impaired responsiveness of the SAN to PNS agonists.

### Effects of CCh on SAN myocyte electrophysiology in db/db mice

**Figure 3:**
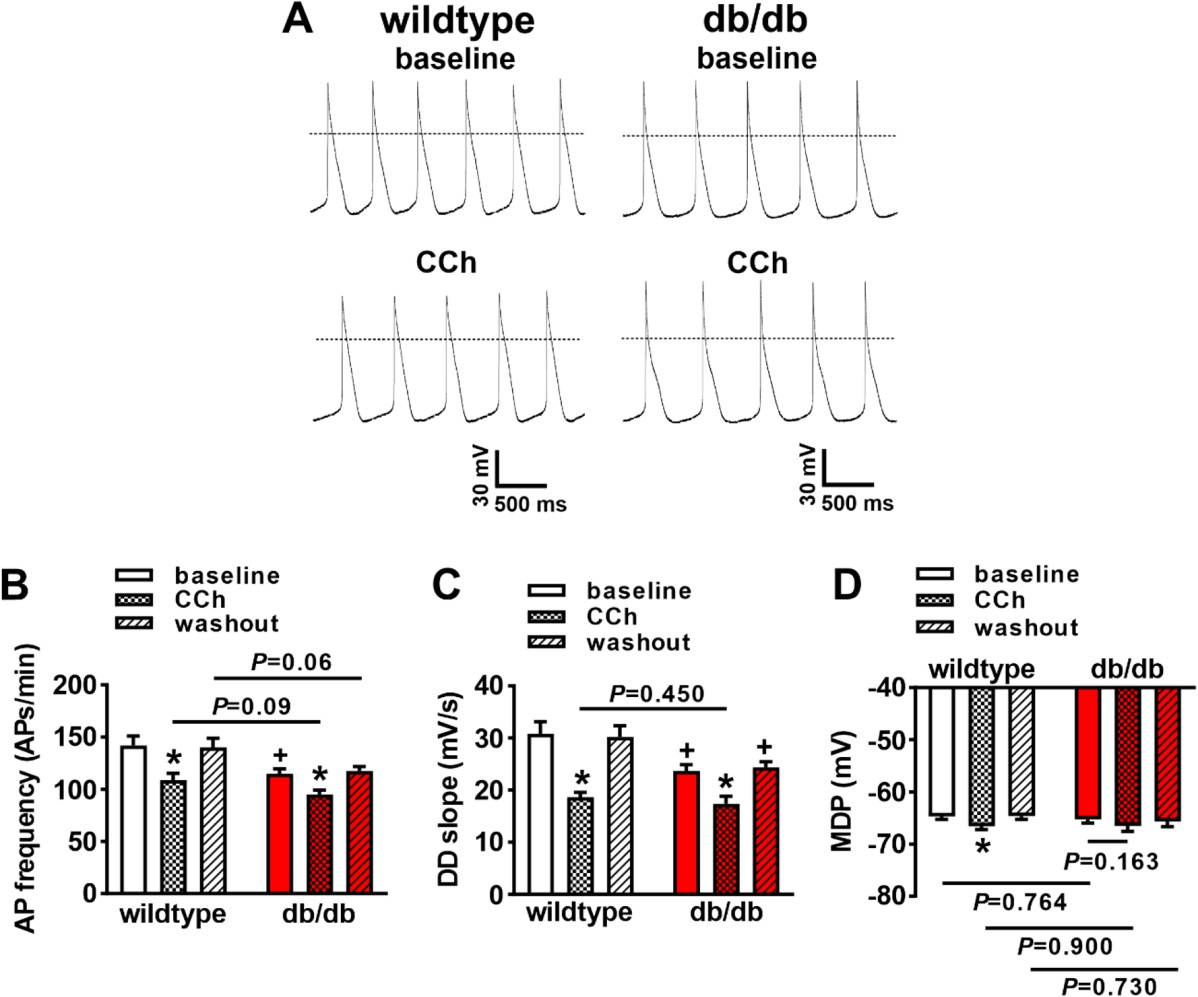
Effects of carbachol on spontaneous action potential firing in isolated sinoatrial node myocytes from db/db mice. (**A**) Representative spontaneous APs at baseline and after CCh (50 nM) in wildtype and db/db SAN myocytes. Dashed lines are at 0 mV. (**B**) Summary of spontaneous AP frequency at baseline and after CCh in wildtype and db/db SAN myocytes. (**C**) Summary of DD slope at baseline and after CCh in wildtype and db/db SAN myocytes. (**D**) Summary of maximum diastolic potential (MDP) at baseline and after CCh in wildtype and db/db SAN myocytes. For panels (**B**–**D**) *n* = 14 wildtype and 13 db/db SAN myocytes; *P < 0.05 vs baseline; ^+^*P* < 0.05 vs wildtype by two-way ANOVA with Holm–Sidak posthoc test.

To determine the basis for the impaired effects of CCh on HR, spontaneous APs were recorded in isolated SAN myocytes from db/db and wildtype mice (Fig. 3A). SAN AP frequency was lower at baseline in db/db mice. CCh (50 nM) reduced SAN AP frequency, but this effect was smaller in db/db SAN myocytes compared to wildtype (Fig. 3B). Consistent with this, CCh elicited a smaller reduction in DD slope (Fig. 3C) and failed to produce a statistically significant hyperpolarization of the MDP (Fig. 3D) in db/db SAN myocytes. These responses were similar in SAN myocytes isolated from male and female db/db and wildtype mice (Supplementary Fig. S1).

**Figure 4:**
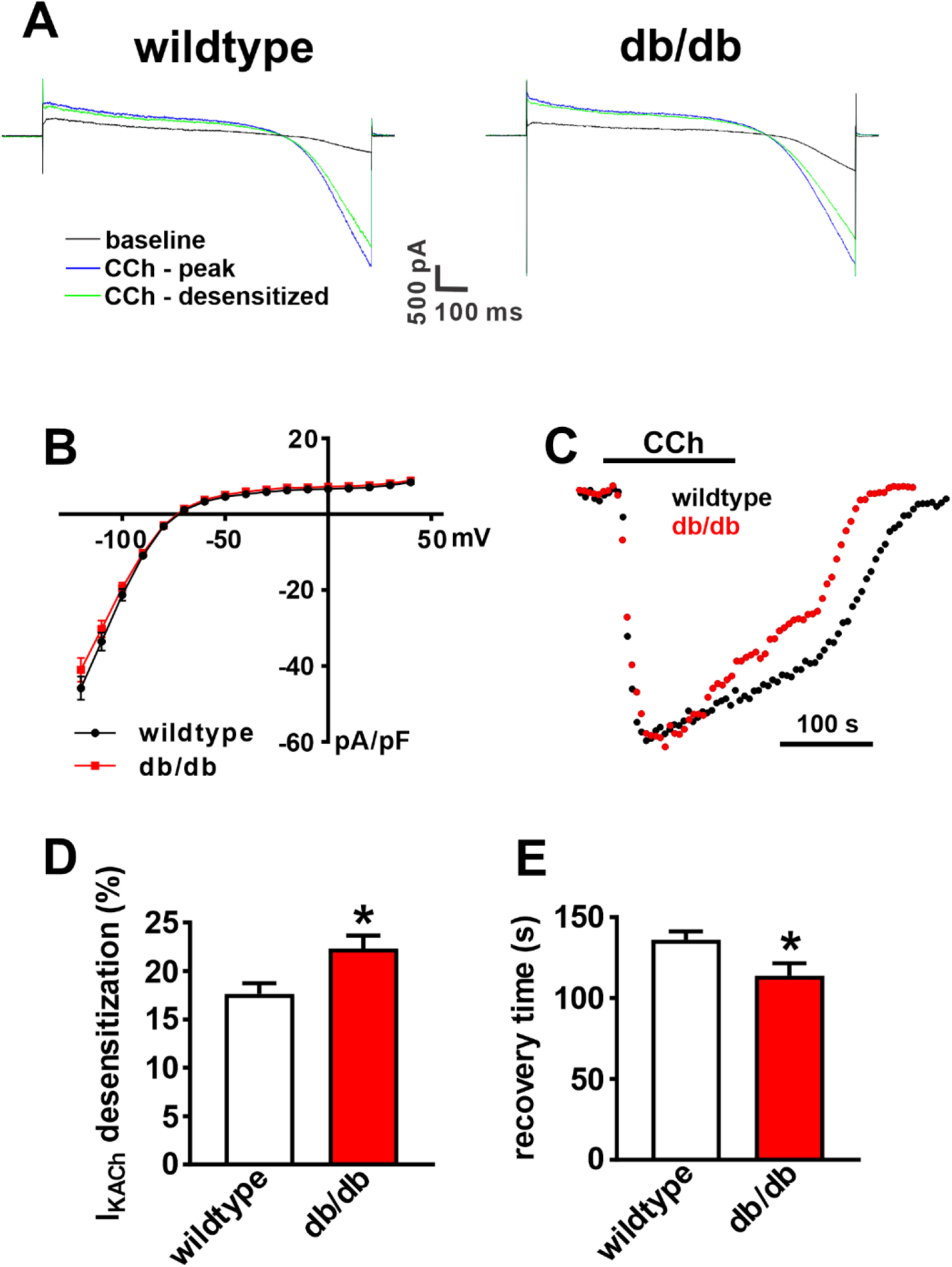
I_KACh_ properties in isolated SAN myocytes from db/db mice. (**A**) Representative I_K_ recordings during a voltage ramp from + 50 mV to − 120 mV (holding potential = − 80 mV) at baseline, at the peak of the CCh (10 µM) response and 2 min after the peak CCh response in wildtype and db/db SAN myocytes. (**B**) Peak I_KACh_ I–V relationship in wildtype (*n* = 28) and db/db (*n* = 25) SAN myocytes. *P* = 0.998 for wildtype vs. db/db by two-way repeated measures ANOVA with Holm–Sidak posthoc test. (**C**) Time course of CCh stimulated I_KACh_, measured at − 100 mV, in wildtype and db/db SAN myocytes. (**D**) Summary of I_KACh_ desensitization in wildtype and db/db SAN myocytes. (**E**) Summary of I_KACh_ recovery time during CCh washout in wildtype and db/db SAN myocytes. For panels (**D**,**E**) *P < 0.05 vs. wildtype by Student’s *t*-test.

The absence of a normal hyperpolarization of the MDP suggests a key role for I_KACh_ in the blunted response to CCh in db/db mice. Thus, I_KACh_ was investigated next. Figure 4A illustrates representative recordings at baseline, at the peak of the CCh (10 µM) response and 2 min after the peak CCh response when I_KACh_ has undergone desensitization. Peak I_KACh_ (i.e. peak of the CCh activated I_K_) was not different between wildtype and db/db SAN myocytes (Fig. 4B). In contrast, plotting the time course of the effects of CCh on I_KACh_ (measured at − 100 mV; Fig. 4C) revealed that I_KACh_ desensitization (i.e. the reduction in I_KACh_ amplitude that occurs in the presence of CCh following peak response) was increased (Fig. 4D) and I_KACh_ recovery time (i.e. deactivation) during CCh washout was faster (Fig. 4E) in db/db SAN myocytes compared to wildtype. There were no differences in expression of *Chrm2* mRNA or M_2_R protein in the SAN in db/db mice (Supplementary Fig. S2). While the mRNA expression of *Kcnj3* was reduced, there were no differences in the expression of *Kcnj5*, or the corresponding proteins, K_ir_3.1 and K_ir_3.4, in the SAN in db/db mice (Supplementary Fig. S3). These data demonstrate that I_KACh_ is impaired in db/db SAN myocytes due to enhanced desensitization and faster deactivation kinetics.

**Figure 5:**
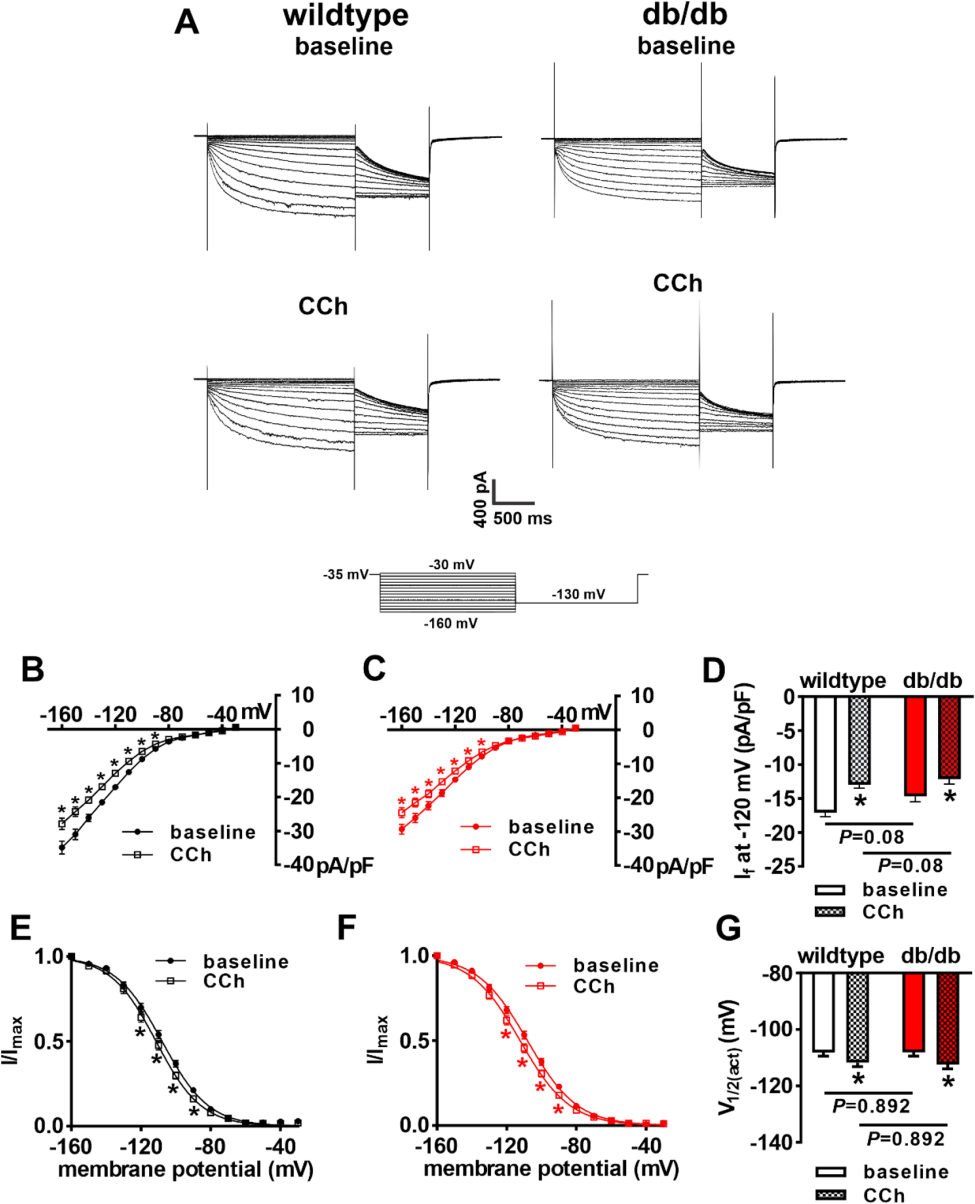
Effects of carbachol on the hyperpolarization-activated current (I_f_) in db/db SAN myocytes. (**A**) Representative I_f_ recordings at baseline and after CCh (10 µM) application in wildtype and db/db SAN myocytes. Voltage clamp protocol shown at bottom of recordings. (**B**) I_f_ IV curves at baseline and after CCh application in wildtype SAN myocytes (*n* = 15). (**C**) I_f_ IV curves at baseline and after CCh application in db/db SAN myocytes (*n* = 16). For panels (**B**) and (**C**) *P < 0.05 vs. baseline by two-way repeated measures ANOVA with Holm–Sidak posthoc test. (**D**) I_f_ density at − 120 mV at baseline and after CCh application in wildtype and db/db SAN myocytes. *P < 0.05 vs baseline by two-way ANOVA with Holm–Sidak posthoc test. (**E**) I_f_ steady-state activation curves at baseline and after CCh application in wildtype SAN myocytes (*n* = 15). (**F**) I_f_ steady-state activation curves at baseline and after CCh application in db/db SAN myocytes (*n* = 16). (**G**) I_f_ V_1/2(act)_ at baseline and after CCh application in wildtype and db/db SAN myocytes. *P < 0.05 vs. baseline by two-way ANOVA with Holm–Sidak posthoc test. Refer to Supplementary Table 1 for additional analysis of I_f_ steady-state activation.

CCh can also reduce SAN AP firing by inhibiting I_f_. Accordingly, the effects of CCh on I_f_ in db/db SAN myocytes were investigated. CCh (10 µm) reduced I_f_ density in wildtype and db/db SAN myocytes (Fig. 5A–C). Comparison of these effects illustrates that I_f_ tended to be smaller at baseline in db/db SAN myocytes and that I_f_ was reduced similarly in both genotypes following application of CCh (Fig. 5D). CCh reduced I_f_ in association with a hyperpolarizing shift in the I_f_ activation curve (Fig. 5E,F). There were no differences in the voltage dependence of activation (V_1/2(act)_) at baseline or after CCh in wildtype and db/db SAN myocytes (Fig. 5G) indicating that the effects of CCh on I_f_ were similar between both groups of mice. There were also no differences in the mRNA expression or protein levels of HCN1, HCN2 or HNC4 in the SAN of db/db mice (Supplementary Fig. S4). These data demonstrate that impaired SAN responsiveness to PNS agonists is not related to altered effects of CCh on I_f_.

Since baseline spontaneous AP firing and DD slope were reduced in db/db SAN myocytes, and this wasn’t accounted for by differences in baseline I_f_, other mechanisms were explored. Repolarizing I_K_ was reduced in db/db SAN myocytes (Supplementary Fig. S5). Furthermore, voltage clamp protocols designed to detect I_Kr_ tail currents revealed that I_Kr_ is reduced in db/db SAN myocytes (Supplementary Fig. S5). This reduction occurred without changes in V_1/2(act)_ or slope factor (k) for I_Kr_ tail currents (Supplementary Fig. S5). I_Kr_ has been shown to be a critical determinant of SAN spontaneous AP firing and DD slope^18^ suggesting that the reduction in I_Kr_ could account for baseline differences in spontaneous AP firing in db/db mice.

### Altered RGS4 and PIP_3_ signaling underlie impaired I_KACh_ in db/db SAN myocytes

**Figure 6:**
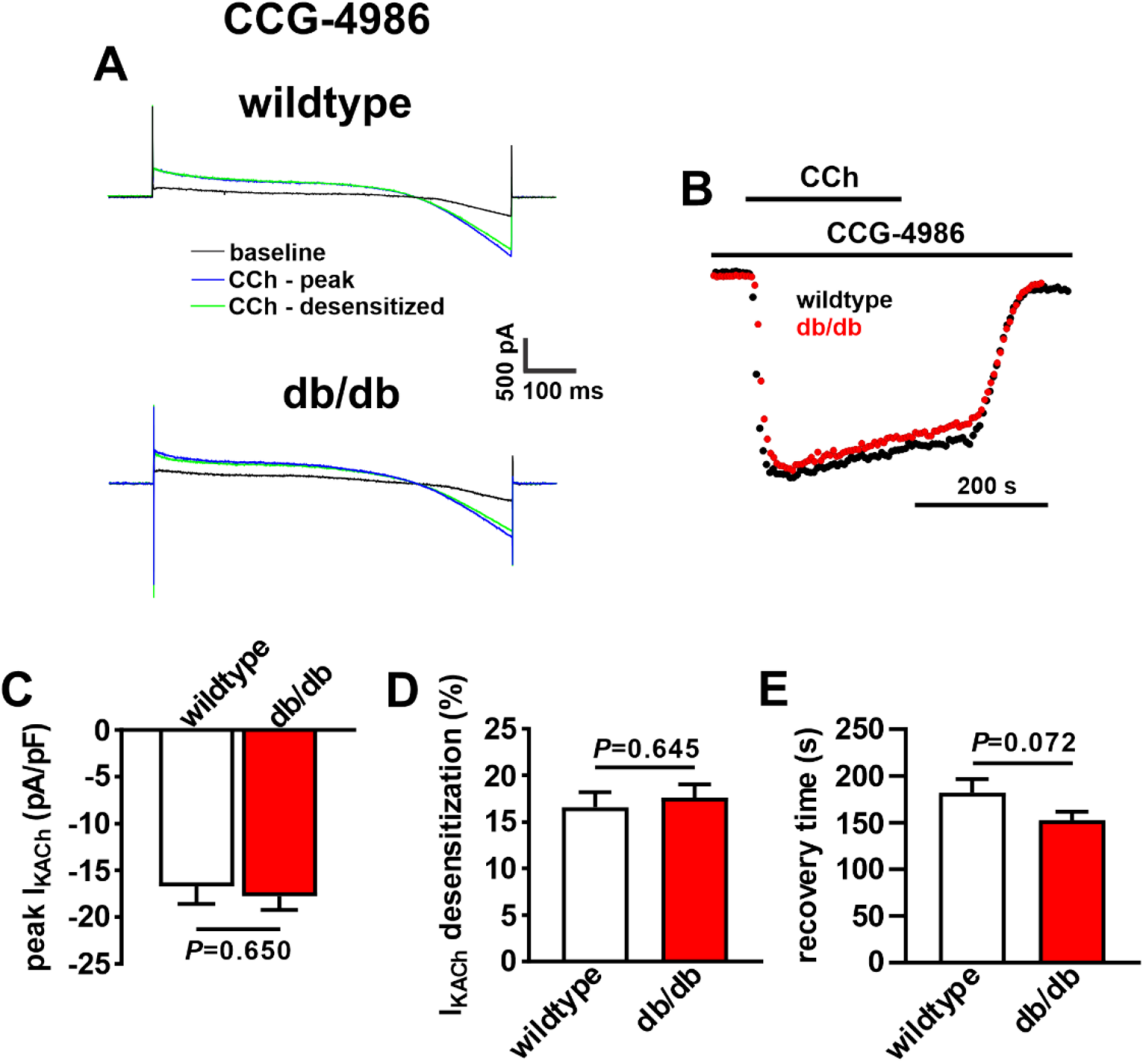
Effects of RGS4 inhibition on I_KACh_ kinetics in db/db SAN myocytes. (**A**) Representative I_K_ recordings illustrating peak and desensitized responses to CCh (10 µM) in wildtype and db/db SAN myocytes pre-treated with the RGS4 inhibitor CCG-4986 (10 µM). (**B**) Time course of CCh stimulated I_KACh_, measured at − 100 mV, in wildtype and db/db SAN myocytes in the presence of CCG-4986. (**C**) Summary of peak I_KACh_ at − 100 mV in wildtype and db/db SAN myocytes in the presence of CCG-4986. (**D**) Summary of I_KACh_ desensitization in wildtype and db/db SAN myocytes in the presence of CCG-4986. (**E**) Summary of I_KACh_ recovery time during CCh washout in wildtype and db/db SAN myocytes in the presence of CCG-4986. For panels (**C**–**E**) *n* = 16 wildtype and 18 db/db SAN myocytes; data analyzed by Student’s *t*-test.

**Figure 7:**
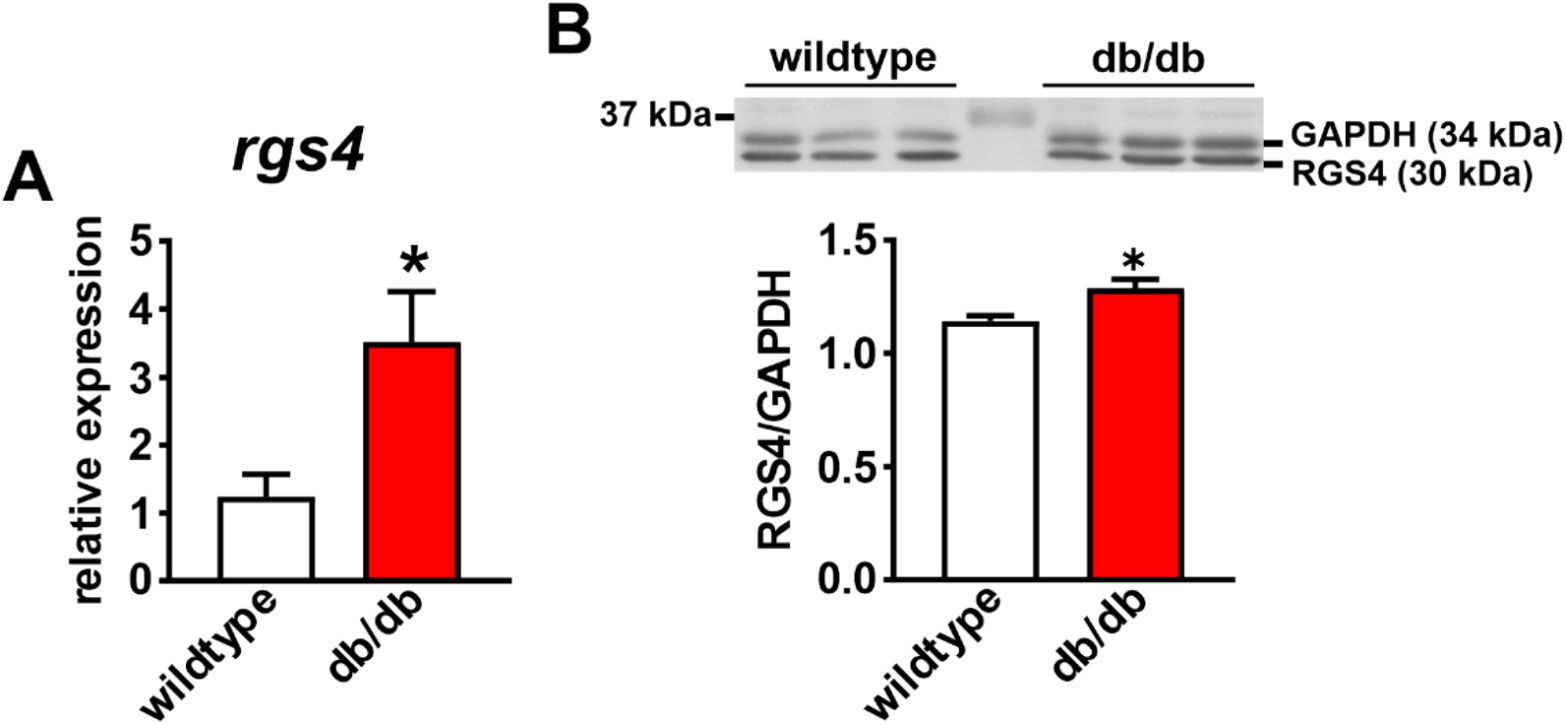
RGS4 gene and protein expression in the SAN in db/db mice. (**A**) mRNA expression of *rgs4* in wildtype (*n* = 5) and db/db (*n* = 5) SAN. *P < 0.05 vs wildtype by Student’s *t*-test. (**B**) Representative Western blot and summary protein expression for RGS4 in the SAN of wildtype (*n* = 6) and db/db (*n* = 6) mice. *P < 0.05 vs. wildtype by Student’s *t*-test. Uncropped Western blot provided in Supplementary Fig. S6.

Previous studies have shown that I_KACh_ desensitization and deactivation kinetics are critically affected by regulator of G protein signaling 4 (RGS4) in the SAN^19^. Furthermore, RGS4 is inhibited by phosphatidylinositol (3,4,5) P_3_ (PIP_3_)^20^ which is activated by insulin-mediated PI3K signaling^21,22^. Therefore, impaired insulin and PI3K signaling in T2DM could result in enhanced RGS4 activity due to a loss of PIP_3_-mediated inhibition of RGS4. To test this hypothesis, wildtype and db/db SAN myocytes were dialyzed with the RGS4 inhibitor CCG-4986 (10 µM) for 10 min prior to studying the effects of CCh (10 µM) on I_KACh_ (Fig. 6A). Time course plots demonstrate that I_KACh_ desensitization and deactivation kinetics were normalized in db/db SAN myocytes after CCG-4986 application (Fig. 6B). Summary data demonstrate that there were no differences in peak I_KACh_ density (Fig. 6C), I_KACh_ desensitization (Fig. 6D) or I_KACh_ recovery time (Fig. 6E) between wildtype and db/db SAN myocytes following inhibition of RGS4 with CCG-4986. RGS4 mRNA expression and protein levels were increased in the SAN in db/db mice (Fig. 7).

**Figure 8:**
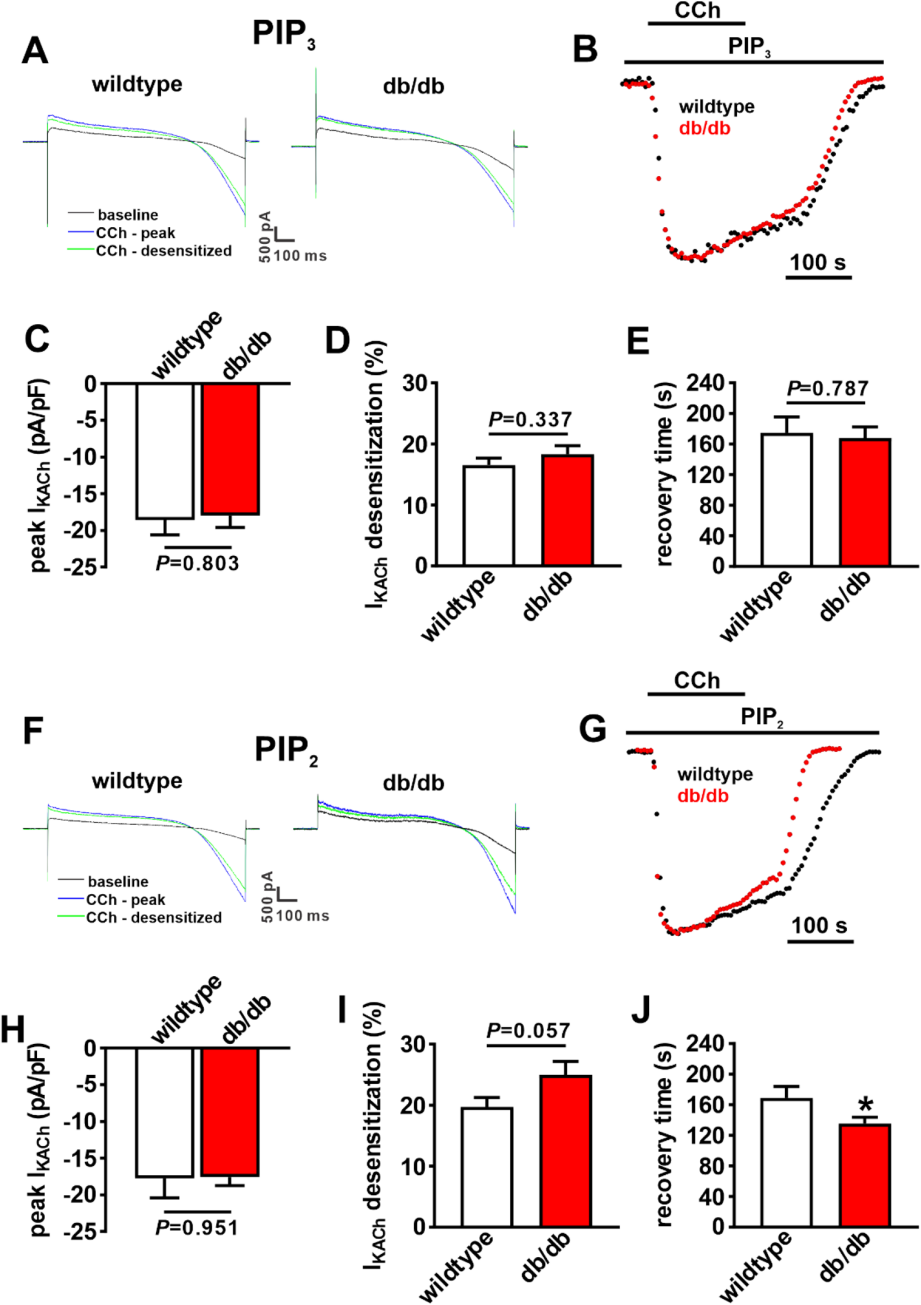
Effects of PIP_3_ on I_KACh_ kinetics in db/db SAN myocytes. (**A**) Representative I_K_ recordings illustrating peak and desensitized responses to CCh in wildtype and db/db SAN myocytes dialyzed with PIP_3_ (1 µM). (**B**) Time course of CCh stimulated I_KACh_, measured at − 100 mV, in wildtype and SAN myocytes dialyzed with PIP_3_. (**C-E **) Summary of peak I_KACh_ at − 100 mV (**C**), I_KACh_ desensitization (**D**) and I_KACh_ recovery time during CCh washoff (**E**) in wildtype (*n* = 16) and db/db (*n* = 19) SAN myocytes dialyzed with PIP_3_. Data in panels (**C**–**E**) analyzed by Student’s *t*-test. (**F**) Representative I_K_ recordings illustrating peak and desensitized responses to CCh in wildtype and db/db SAN myocytes dialyzed with PIP_2_ (1 µM). (**G**) Time course of CCh stimulated I_KACh_, measured at − 100 mV, in wildtype and SAN myocytes dialyzed with PIP_2_. (**H**–**J**) Summary of peak I_KACh_ at − 100 mV (**H**), I_KACh_ desensitization (**I**) and I_KACh_ recovery time during CCh washoff (**J**) in wildtype (*n* = 18) and db/db (*n* = 19) SAN myocytes dialyzed with PIP_2_. For panels (**H**–**J**) *P < 0.05 by Student’s *t*-test.

Next, wildtype and db/db SAN myocytes were dialyzed with PIP_3_ (1 µM) for 10 min prior to application of CCh (10 µM) to investigate I_KACh_ properties (Fig. 8A). Similar to RGS4 inhibition, time course plots demonstrate that I_KACh_ desensitization and deactivation were normalized in db/db SAN myocytes treated with PIP_3_ (Fig. 8B). On average, peak I_KACh_ density (Fig. 8C), I_KACh_ desensitization (Fig. 8D) and I_KACh_ recovery time (Fig. 8E) were not different between wildtype and db/db SAN myocytes when treated with PIP_3_.

In control experiments, wildtype and db/db SAN myocytes were dialyzed with phosphatidylinositol 4,5-bisphosphate (PIP_2_), a phospholipid that does not directly inhibit RGS4 or mediate the actions of insulin-dependent PI3K signaling. PIP_2_ (1 µM) was dialyzed into SAN myocytes for 10 min prior to application of CCh to investigate I_KACh_ (Fig. 8F). Time course plots demonstrate that in the presence of PIP_2_, I_KACh_ desensitization remained enhanced and I_KACh_ deactivation was faster in db/db SAN myocytes (Fig. 8G). Summary data show that in the presence of PIP_2_, peak I_KACh_ density was not different (Fig. 8H), but that I_KACh_ desensitization tended to be increased (Fig. 8I) and recovery time was faster (Fig. 8J) in db/db SAN myocytes compared to wildtype.

## Discussion

The present study demonstrates that db/db mice display impaired responsiveness to the PNS agonist CCh in the SAN leading to impaired HR regulation, indicating that this mouse model of T2DM reproduces the phenotype of impaired ANS regulation of the heart seen in T2DM patients. Furthermore, the present study provides novel insight into the cellular and molecular mechanisms leading to blunted PNS regulation of HR in T2DM. In isolated SAN myocytes, CCh displayed a reduced ability to slow spontaneous AP firing in association with a smaller reduction in DD slope and less hyperpolarization of the MDP in db/db mice. In fact, CCh failed to produce a statistically significant hyperpolarization in db/db SAN myocytes. These responses were similar between male and female db/db mice.

Consistent with these effects, I_KACh_ was centrally involved in the impaired effects of CCh on AP firing in SAN myocytes. Analysis of I_KACh_ properties revealed that the peak I_KACh_ density was not altered in db/db SAN myocytes. Similarly, there were no major changes in gene or protein expression of M_2_R or K_ir_3 channels in the SAN in db/db mice. Rather, the major alterations in I_KACh_ in db/db SAN myocytes were an increase in desensitization and faster deactivation kinetics. These findings are consistent with our studies in vivo, which demonstrated that the effects of CCh were reduced in db/db mice (likely in association with enhanced desensitization of I_KACh_) and that reversal of the effects of CCh upon application of atropine was much more rapid in db/db mice (likely in association with faster I_KACh_ deactivation). Both of these effects resulted in impaired PNS effects on HR (and SAN function) over the full time-course of CCh application and removal. Our findings are also consistent with previous studies demonstrating that I_KACh_ plays a critical role in mediating SAN and HR responses to CCh^23,24^.

I_KACh_ is well known to undergo a process of desensitization whereby current amplitude decreases in the presence of M_2_R agonists such as CCh^25–27^. I_KACh_ desensitization, as well as deactivation are critically affected by regulator of G protein signaling (RGS) proteins, including RGS4, which is highly expressed in the SAN^19,28^. Genetic ablation of RGS4 leads to enhanced PNS signaling due to reductions in I_KACh_ desensitization and slower I_KACh_ deactivation^19^. Thus, increased I_KACh_ desensitization and faster deactivation in db/db SAN myocytes (i.e. the opposite of RGS4 ablation) suggested enhanced RGS4 activity in db/db mice. This was confirmed using CCG-4986, which is a selective RGS4 inhibitor at the doses used in this study^29,30^. These findings are consistent with previous studies demonstrating an essential role for RGS proteins in regulated PNS signaling in the heart via effects on I_KACh_^19,28,31–33^.

RGS4 is importantly regulated by PIP_3_, which exerts an inhibitory effect on RGS4 activity^20,34,35^. In the normal heart, PIP_3_ is generated from PIP_2_ through the actions of PI3Kα (p100α isoform)^21^. In T2DM, impaired insulin signaling results in reduced PI3K activity, which would result in less PIP_3_ generation^22,36^. This loss of PIP_3_ would lead to less inhibition of RGS4 and could underlie increased RGS4 activity in the SAN of db/db mice. In support of this, bypassing insulin signaling and directly dialyzing db/db SAN myocytes with PIP_3_ normalized I_KACh_ desensitization and deactivation. Thus, the studies conducted here demonstrate that the kinetic properties of I_KACh_ are altered in T2DM due to the loss of PIP_3_ mediated inhibition of RGS4 and that this is a major determinant of impaired PNS signaling to the SAN and HR regulation in T2DM.

CCh can also reduce spontaneous AP firing by inhibiting I_f_, which was also investigated in the present study. As expected, CCh reduced I_f_ density in association with a hyperpolarizing shift in the V_1/2(act)_; however, this effect was similar in wildtype and db/db SAN myocytes. This indicates that HCN channel function was not altered by a change in RGS4 activity in T2DM. CCh can also modulate SAN activity via other mechanisms such as L-type Ca^2+^ currents and SR Ca^2+^ handling^1,6^. These targets were not investigated in this study; therefore, whether the effects of CCh on these targets are altered in db/db mice and whether these targets are regulated by RGS4 is presently unknown. These could be areas for future study. Nevertheless, the absence of altered CCh effects on I_f_ (which is also an important mediator of PNS signaling in the SAN) in db/db mice suggests that the effects of RGS4 are selective for K_ir_3 channels. In addition to RGS4, RGS6 has also been shown to regulate I_KACh_ kinetics in the SAN^33,37^. Whether RGS6 is regulated by PIP_3_ in similar ways to RGS4 and whether RGS6 contributes to impaired PNS signaling in DM is not currently known.

PNS signaling in the SAN has also been previously investigated in T1DM using Akita mice^8^. The present study demonstrates that while there are similarities in how PNS signaling is altered in Akita and db/db mice, there are also important differences. Similar to the present study in db/db mice, HR regulation by CCh was impaired in Akita mice in association with impaired responsiveness of SAN myocytes to CCh. I_KACh_ desensitization and deactivation also showed similar changes in Akita SAN myocytes to those identified here in db/db mice and these changes were reversible by RGS4 inhibition or application PIP_3_ in both models of DM. Conversely, Akita mice showed no differences in expression of RGS4 in the SAN^8^, whereas RGS4 gene and protein expression were increased in the SAN in db/db mice. This indicates that RGS4 plays a central role in impaired PNS signaling in the SAN in T1DM and T2DM, but that there are some differences in how RGS4 is altered in the two forms of DM. The increase in RGS4 expression, in combination with increased RGS4 activity due to loss of PIP_3_ signaling, demonstrates that alterations in RGS4 are more complex and multifaceted in db/db mice compared to Akita mice. This could explain, at least in part, the finding that CAN often develops earlier in T2DM compared to T1DM patients^4^. Collectively, these data demonstrate that RGS4 signaling is particularly important in the SAN in type 2 diabetic db/db mice due to multiple alterations including increases in its gene and protein expression as well as its functional regulation by PI3K-PIP_3_ signaling.

The present study also shows that baseline HR and spontaneous AP firing in SAN myocytes are reduced in db/db mice. This is consistent with previous studies showing lower HR and impaired SAN function in animal models of T1DM and T2DM^12,38–42^. In the present study, baseline I_f_ density tended to be lower in db/db SAN myocytes; however, there were no differences in baseline V_1/2(act)_ or in the gene and protein expression of HCN1, HCN2 or HCN4 in db/db mice suggesting I_f_ does not play a major role in baseline HR differences. I_Kr_ plays an integral role in the SAN where it controls AP repolarization, contributes to the DD slope and helps determine AP firing frequency^18^. We measured I_Kr_ from tail currents, which showed V_1/2(act)_ values very similar to those reported in previous studies for this current^18^, and found that I_Kr_ is reduced in db/db SAN myocytes, suggesting that this could contribute to baseline differences in HR and SAN activity in db/db mice. Additional studies will be required to investigate the roles of I_f_, I_Kr_ and other ionic mechanisms not assessed in this study in baseline differences in SAN AP firing in db/db mice. Reduced basal HR in vivo in the presence of impaired PNS signaling suggests additional alterations in db/db mice. Baseline HR is determined by intrinsic SAN function, the balance between sympathetic and parasympathetic nervous system regulation of the SAN and other factors such as circulating factors that affect the SAN. Our study suggests that even though PNS signaling is impaired, baseline HR is reduced in part due to impaired intrinsic SAN function. Furthermore, sympathetic nervous system activity was not investigated in this study, but may also be altered in db/db mice. As such, future studies investigating sympathetic regulation of HR and baseline HR differences in db/db mice are warranted.

In summary, the present study provides new insight into the basis for impaired PNS regulation of HR in T2DM. The effects of CCh on HR were reduced in association with impaired responsiveness of SAN myocytes to CCh. I_KACh_, which is a key mediator of the effects of PNS activation in the SAN, displayed enhanced desensitization and faster deactivation kinetics in db/db SAN myocytes resulting in blunted effects of CCh on HR throughout the duration of CCh exposure and removal. These I_KACh_ alterations occurred due to enhanced RGS4 activity and the loss of PIP_3_ signaling in db/db SAN myocytes. These findings, which identify potential new targets for intervention in diabetic patients, should be taken into consideration when interpreting CAN and blunted ANS signaling to the heart in T2DM.

## Supplementary Information

Supplementary Information.

## Data Availability

Any data or experimental reagents are available from the corresponding author upon reasonable request.
