# Supplementary material for: Impaired regulation of heart rate and sinoatrial node function by the parasympathetic nervous system in type 2 diabetic mice
Source: Sci Rep. 2021 Jun 14;11:12465. doi: 10.1038/s41598-021-91937-2 (PMC8203800; doi:10.1038/s41598-021-91937-2)
Supplement: Supplementary file 1 — Supplementary Information. [file 41598_2021_91937_MOESM1_ESM.pdf]

Supplemental Material

Impaired regulation of heart rate and sinoatrial node function by the parasympathetic nervous system in type 2 diabetic mice

Yingjie Liu[a], Hailey J. Jansen[a], Pooja S. Krishnaswamy[b], Oleg Bogachev[b], Robert A. Rose[a*]

[a]Libin Cardiovascular Institute
Department of Cardiac Sciences
Department of Physiology and Pharmacology
Cumming School of Medicine
University of Calgary
Calgary, Alberta, Canada

[b]Department of Physiology and Biophysics
Dalhousie University
Halifax, Nova Scotia, Canada

Supplemental Methods

Animals

Male and female littermate wildtype and db/db mice[1] between the ages of 16 and 20 weeks were used in this study. This mouse contains an autosomal recessive mutation in the leptin receptor (*Lepr*) gene. Mice homozygous for the mutation display hyperphagia and hyperglycemia[2]. Db/db mice were initially obtained from the Jackson Laboratory (strain C57BL/6J-*Lepr*^{db}) and then bred locally. Homozygous db/db mice and wildtype littermates were used in this study. Animals were fed *ad libitum* and kept on a 12:12 h light-dark cycle. At the age range used in this study db/db mice were overtly obese and hyperglycemic as we have shown previously[3].

Intracardiac electrophysiology and electrocardiogram recording

Surface ECGs (used to assess changes in heart rate) were measured in anesthetized mice (2% isoflurane inhalation) using 30 gauge subdermal needle electrodes (Grass Technologies). A 1.2 french octapolar electrophysiology catheter containing 8 electrodes spaced 0.5 mm apart (Transonic) was used for intracardiac pacing experiments. Correct catheter placement was ensured by obtaining a ventricular signal in the distal lead and a predominant atrial signal in the proximal lead. Sinoatrial node recovery time (SNRT) was measured by delivering a 12 stimulus drive train at a cycle length of 100 ms. SNRT is defined as the time between the last stimulus in the drive train and the occurrence of the first spontaneous atrial beat (P wave). SNRT was corrected for heart rate (cSNRT) by subtracting the prestimulus RR interval from the measured SNRT. All ECG data were acquired using a Gould ACQ-7700 amplifier and Ponemah Physiology Platform software (Data Sciences International) as we have described previously[4,5]. Body temperature was maintained at 37˚C using a heating pad. CCh and atropine were delivered by intraperitoneal injection.

Isolation of mouse sinoatrial node myocytes

Mouse SAN myocytes were isolated using procedures we have described previously[6-8]. Mice were administered a 0.2 ml intraperitoneal injection of heparin (1000 IU/ml) to prevent blood clotting. Following this, mice were anesthetized by isoflurane inhalation and then sacrificed by cervical dislocation. The heart was excised into Tyrode's solution (35ºC) consisting of (in mM) 140 NaCl, 5.4 KCl, 1.2 KH_2PO_4, 1.0 $MgCl_2$, 1.8 $CaCl_2$, 5.55 glucose, and 5 HEPES, with pH adjusted to 7.4 with NaOH. Atrial preparations were dissected and the SAN region was cut into strips, which were transferred and rinsed in a 'low Ca^{2+}, Mg^{2+} free' solution containing (in mM) 140 NaCl, 5.4 KCl, 1.2 KH_2PO_4, 0.2 $CaCl_2$, 50 taurine, 18.5 glucose, 5 HEPES and 1 mg/ml bovine serum albumin (BSA), with pH adjusted to 6.9 with NaOH. SAN tissue strips were digested in 5 ml of 'low Ca^{2+}, Mg^{2+} free' solution containing collagenase (type II, Worthington Biochemical Corporation), elastase (Worthington Biochemical Corporation) and protease (type XIV, Sigma Chemical Company) for 30 min. The tissue was then transferred to 5 ml of modified KB solution containing (in mM) 100 potassium glutamate, 10 potassium aspartate, 25 KCl, 10 KH_2PO_4, 2 $MgSO_4$, 20 taurine, 5 creatine, 0.5 EGTA, 20 glucose, 5 HEPES, and 0.1% BSA, with pH adjusted to 7.2 with KOH. The tissue was mechanically agitated using a wide-bore pipette. This procedure yielded individual SAN myocytes with cellular automaticity that was recovered after readapting the cells to a physiological concentration of Ca^{2+}. SAN myocytes were identified by their small spindle shape and ability to beat spontaneously in the recording chamber when superfused with normal Tyrode's solution. When patch-clamped, SAN myocytes always displayed spontaneous action potentials. The capacitance of single SAN myocytes was 20 – 35 pF.

Solutions and electrophysiological protocols

Spontaneous action potentials, as well as the acetylcholine activated K^+ current (I_{KACh}), the hyperpolarization activated current (I_f) and the rapid delayed rectifier K^+ current (I_{Kr}) were recorded in single SAN myocytes using the patch-clamp technique in the whole cell configuration[9,10]. Action potentials and ionic currents were recorded at room temperature (22-23°C). I_{KACh} was investigated using a voltage ramp from +50 mV to -120 mV (holding potential was -80 mV) before and after application of carbachol (CCh; 10 µM) as we have described previously[10]. I_{KACh} was quantified as the CCh-sensitive difference current. I_f was recorded by applying a series of 2.5 s voltage clamp steps between -150 mV and -30 mV, followed by a step to -130 mV, from a holding potential of -35 mV. Activation kinetics for I_f were determined by normalizing tail currents at each voltage to the maximum current level at -130 mV and fitting the data to the Boltzmann function: $I/I_{max}=1/(1+exp[(V_m-V_{1/2})/k])$ where V_m is the potential of the voltage clamp step, $V_{1/2}$ is the voltage at which 50% activation occurs and k is the slope factor. I_{Kr} was recorded by applying a series of 1 s voltage clamp steps between -50 and +50 mV, followed by a 2 s step to -45 mV to elicit I_{Kr} tail currents[11]. I_{Kr} tail currents were fit to the Boltzmann function: $I=I_{max}/(1+exp(V_m-V_{1/2})/k)$ where V_m is the potential of the voltage clamp step, $V_{1/2}$ is the voltage at which 50% activation occurs and k is the slope factor[11].

For recording APs, I_{KACh}, I_{Kr} and I_f, the recording chamber was superfused with a normal Tyrode's solution (22 – 23ºC) containing (in mM) 140 NaCl, 5 KCl, 1 $MgCl_2$, 1 $CaCl_2$, 10 HEPES, and 5 glucose, with pH adjusted to 7.4 with NaOH. The pipette filling solution for APs, I_{KACh}, I_{Kr} and I_f, contained (in mM) 135 KCl, 0.1 $CaCl_2$, 1 $MgCl_2$, 5 NaCl, 10 EGTA, 4 Mg-ATP, 6.6 Na-phosphocreatine, 0.3 Na-GTP and 10 HEPES, with pH adjusted to 7.2 with KOH. 2 mM $BaCl_2$ was added to the superfusate when recording I_f in order to block I_{KACh}[12].

Micropipettes were pulled from borosilicate glass (with filament, 1.5 mm OD, 0.75 mm ID, Sutter Instrument Company) using a Flaming/Brown pipette puller (model p-87, Sutter Instrument Company). The resistance of these pipettes was 4 – 8 MΩ when filled with recording solution. Micropipettes were positioned with a micromanipulator (Burleigh PCS-5000 system)

mounted on the stage of an inverted microscope (Olympus IX71). Seal resistance was 2 – 15 GΩ. Rupturing the sarcolemma in the patch experiments resulted in access resistances of 5 – 15 MΩ. Series resistance compensation averaged 80 – 85% using an Axopatch 200B amplifier (Molecular Devices). Data were digitized using a Digidata 1440 and pCLAMP 10 software (Molecular Devices) and stored on computer for analysis.

Spontaneous AP parameters, including the maximum diastolic potential (MDP), the slope of the diastolic depolarization (DD slope) and the take-off potential were analyzed as described previously[13-15]. The DD slope was measured by fitting a straight line to the initial linear portion (~2/3) of this AP component as we and other have previously done[6,7,14,16].

Quantitative PCR

Quantitative gene expression was measured in the SAN as we have described previously[4,6,17]. Intron-spanning primers were designed for *Chrm2* (M_2R), *Kcnj3* ($K_{ir}3.1$), *Kcnj5* ($K_{ir}3.4$), *Hcn1*, *Hcn2*, and *Hcn4*. Glyceraldehyde 3-phosphate dehydrogenase (GAPDH) was used as a reference gene. Primer sequences are listed in Supplemental Table 2 below.

Total RNA was isolated from the SAN using a PureZOL™ RNA Isolation Reagent and the Aurum™ Total RNA Fatty and Fibrous Tissue Kit (Bio-Rad Laboratories) as per kit instructions. RNA samples were eluted from the spin column in 40 µL elution buffer. RNA yield and purity were assessed using a Nanodrop. All samples had a A_{260}/A_{280} of over 2.0 and therefore were free of DNA contamination. Next, cDNA (2.5 ng/µL) was synthesized using the iScript™ cDNA Synthesis Kit (Bio-Rad Laboratories). Reactions were performed in a Bio-Rad MyCycler thermal cycler using the following protocol: 5 min of priming at 25ºC followed by reverse transcription for 30 min at 42ºC then 5 min at 85ºC to inactivate reverse transcriptase.

All qPCR reactions were run in duplicate in 10 µL reactions that contained the following: 4 µL sample cDNA, 5.6 µL GoTaq® qPCR Master Mix (Promega), and 0.4 µL primers. Primers were reconstituted to a final concentration of 100 µM with nuclease free water and stored at -

20ºC until use. Primers were diluted to 10 µM for qPCR reactions. RT-qPCR reactions were performed using the CFX96 Touch™ Real-Time PCR Detection System (Bio-Rad) using the following protocol: Taq polymerase was activated for 2 min at 95ºC followed by 39 cycles of denaturing for 15 s at 95ºC, annealing for 30 s at 60ºC, and extension for 30s at 72ºC. This was followed by melt curve analysis from 65-95ºC in 0.5ºC increments. Data were analyzed using the $2^{-\Delta\Delta C_T}$ method using the CFX Manager Software version 3.1 (Bio-Rad). Gene expression was normalized to GAPDH.

Western blotting

Protein samples were extracted from two SANs and pooled for each experimental replicate in order to ensure sufficient protein, as we[6] and others[18] have described previously. Tissues were pre-cooled in liquid nitrogen and homogenized in an ice-cold RIPA buffer (50mM Tris, 150mM NaCl, 1mM EDTA, 25mM sucrose, 1% Triton, 0.1% SDS) containing 0.5mM DTT (1,4-Dithiothreitol, Roche) and Protease Inhibitor Cocktail (Sigma-Aldrich). Preparation was centrifuged at 10000 rpm at 4^{0}C for 10 min and supernatant was collected. Protein concentrations were measured using a Bio-Rad DC Protein Assay Kit II (Bio-Rad). Protein samples (20 µg/lane) were prepared in Laemmli sample buffer (Bio-Rad) containing 355 mM 2-marcaptoethanol (Bio-Rad) separated by 7.5% SDS-polyacrylamide gels (SDS-PAGE) and transferred onto Biotrace™ NT nitrocellulose Transfer Membrane (VWR). The membrane was blocked with 1% casein in tris-buffered saline (TBS; Bio-Rad) for 1 hour and incubated overnight at 4^{0}C with rabbit primary antibodies separately including HCN1 1:500 (Alomone Labs), HCN2 1:500 (Alomone Labs), HCN4 1:500 (Alomone Labs), M_2R 1:500 (Abcam), $K_{ir}3.1$ 1:1000 (Abcam), $K_{ir}3.4$ 1:1000 (Abcam) and RGS4 1:500 (Aviva Systems Biology). The membrane was washed 3 times with TBST (TBS with 1% Tween 20 (Bio-Rad)) and incubated with StarBright700 anti-Rabbit secondary antibody (Bio-Rad) at 1:10000 together with Rhodamine anti-GAPDH at 1:5000 (Bio-Rad) for 1 hour at room temperature. Then the membrane was

washed again 3 times with TBST and scanned using the ChemiDoc system (Bio-Rad). We quantified expression of each of these proteins based on the identification of bands at the predicted molecular weight according to the information from the supplier.

Supplemental Figures

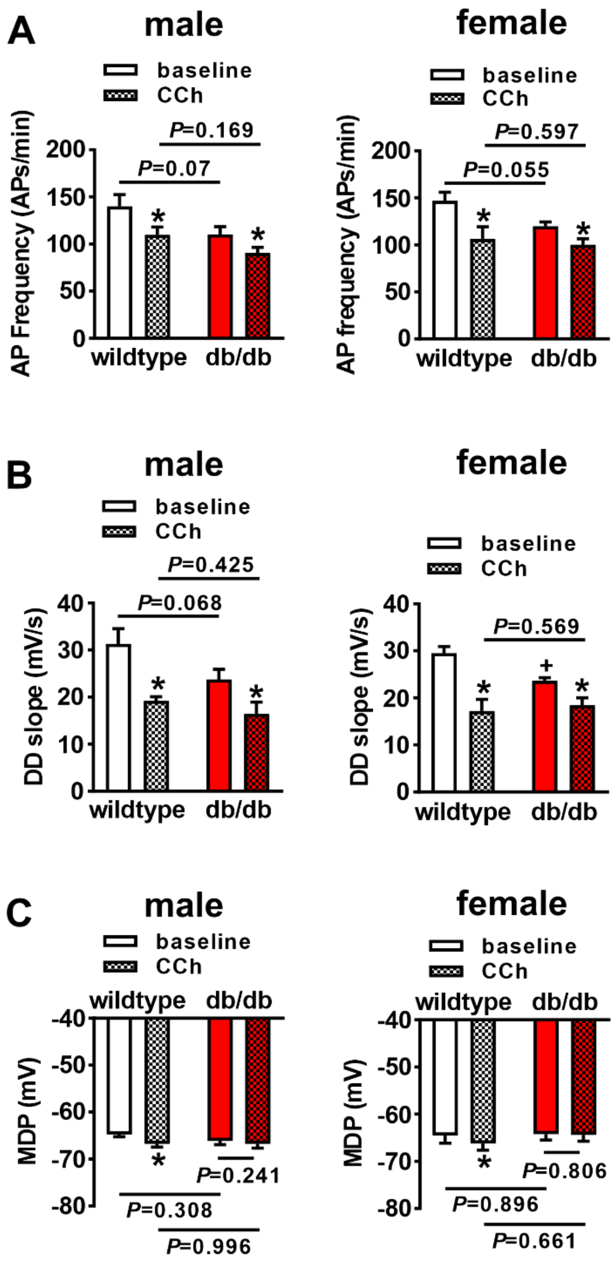

Figure S1: Comparison of spontaneous action potential parameters in isolated sinoatrial node myocytes from male and female db/db mice. A: Summary of spontaneous AP frequency at baseline and after CCh (50 nM) in male and female SAN myocytes from wildtype and db/db mice. **B:** Summary of DD slope at baseline and after CCh (50 nM) in male and female SAN myocytes from wildtype and db/db mice. **C:** Summary of MDP at baseline and after CCh (50 nM) in male and female SAN myocytes from wildtype and db/db mice. For all panels *n*=10 wildtype male cells, 7 db/db male cells, 4 wildtype female cells and 6 db/db female cells. **P*<0.05 vs. baseline; $^{+}$*P*<0.05 vs wildtype by two way repeated measures ANOVA with Holm-Sidak posthoc test.

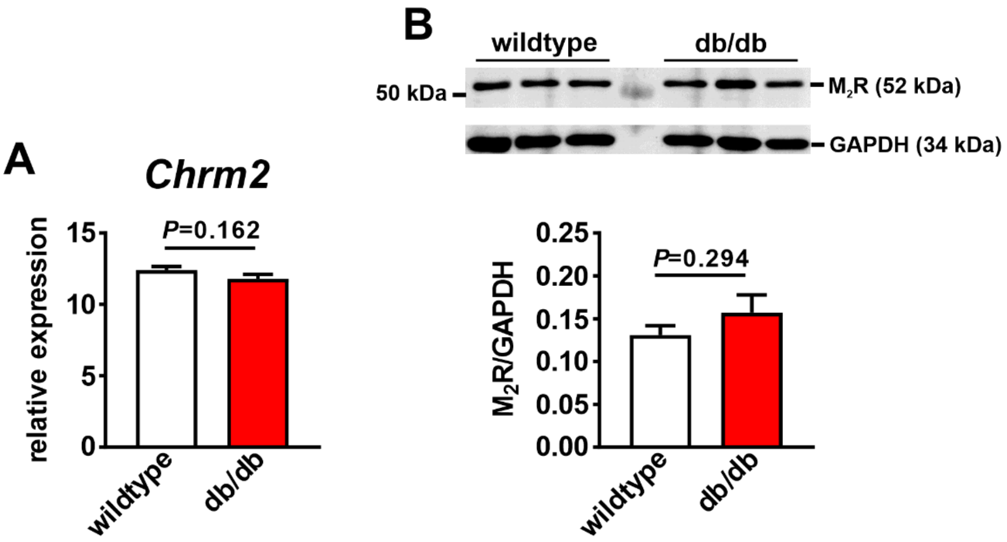

Figure S2: Muscarinic (M_2) receptor gene and protein expression in the SAN in db/db mice. **A:** mRNA expression of *Chrm2* in wildtype (*n*=8) and db/db (*n*=8) SAN. Data analyzed by Student's *t*-test. **B:** Representative Western blot and summary protein expression for M_2 receptor (M_2R) in wildtype (*n*=6) and db/db (*n*=6) SAN. Data analyzed by Student's *t*-test. Uncropped Western blot provided Supplemental Fig. S6.

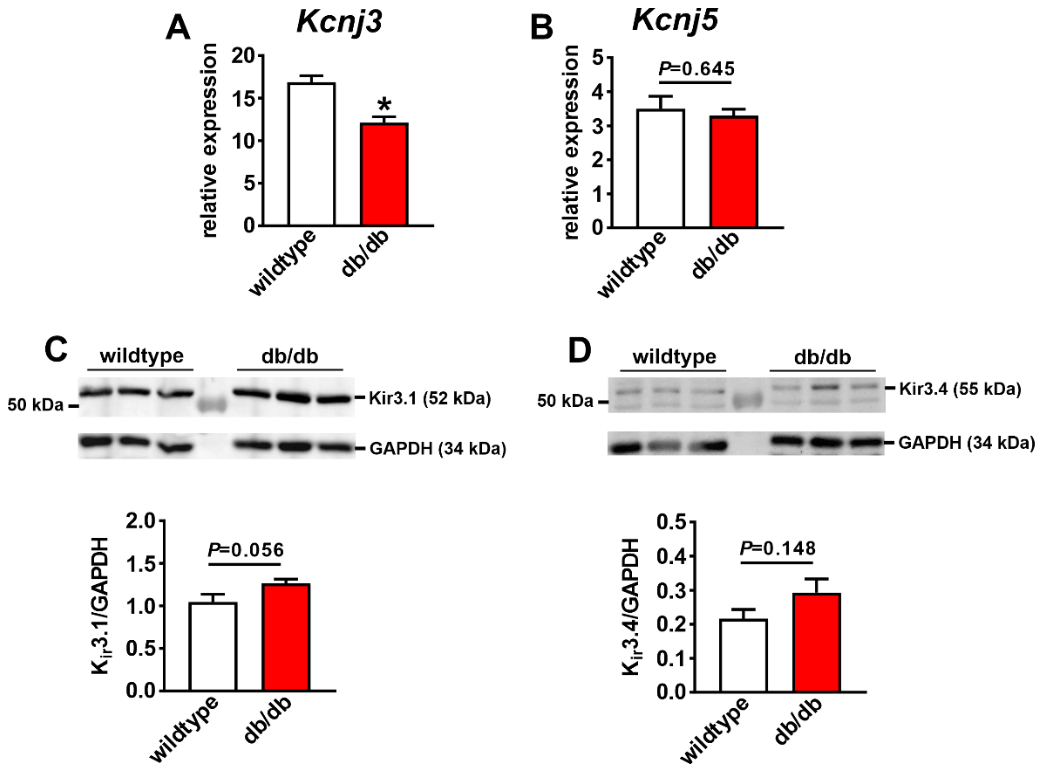

Figure S3: Gene and protein expression of I_{KACh} subunits in the SAN in db/db mice. **A and B:** mRNA expression of *Kcnj3* (**A**) and *Kcnj5* (**B**) in wildtype (*n*=8) and db/db (*n*=8) SAN. **P*<0.05 vs wildtype by Student's *t*-test. **C and D:** Representative Western blots and summary protein expression for $K_{ir}3.1$ (**C**) and $K_{ir}3.4$ (**D**) in wildtype (*n*=6) and db/db (*n*=6) SAN. Data analyzed by Student's *t*-test. Uncropped Western blots provided Supplemental Fig. S6.

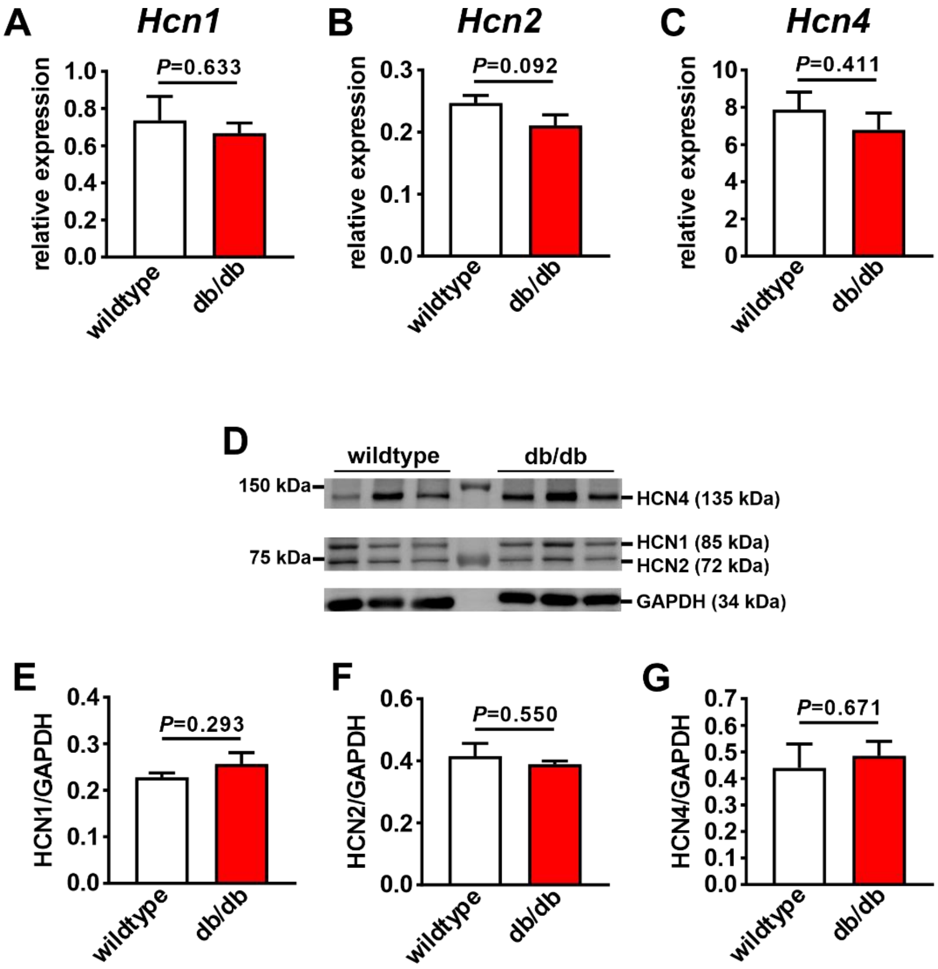

Figure S4: Gene and protein expression of I_f subunits in the SAN in db/db mice. **A-C:** mRNA expression of *Hcn1* (**A**), *Hcn2* (**B**) and *Hcn4* (**C**) in wildtype (*n*=16) and db/db (*n*=14) SAN. Data analyzed by Student's *t*-test. **D:** Representative Western blot for HCN1, HCN2 and HCN4. **E-G:** Summary protein expression for HCN1 (**E**), HCN2 (**F**) and HCN4 (**G**) in wildtype (*n*=6) and db/db (*n*=6) SAN. Data analyzed by Student's *t*-test. Uncropped Western blot provided Supplemental Fig. S6.

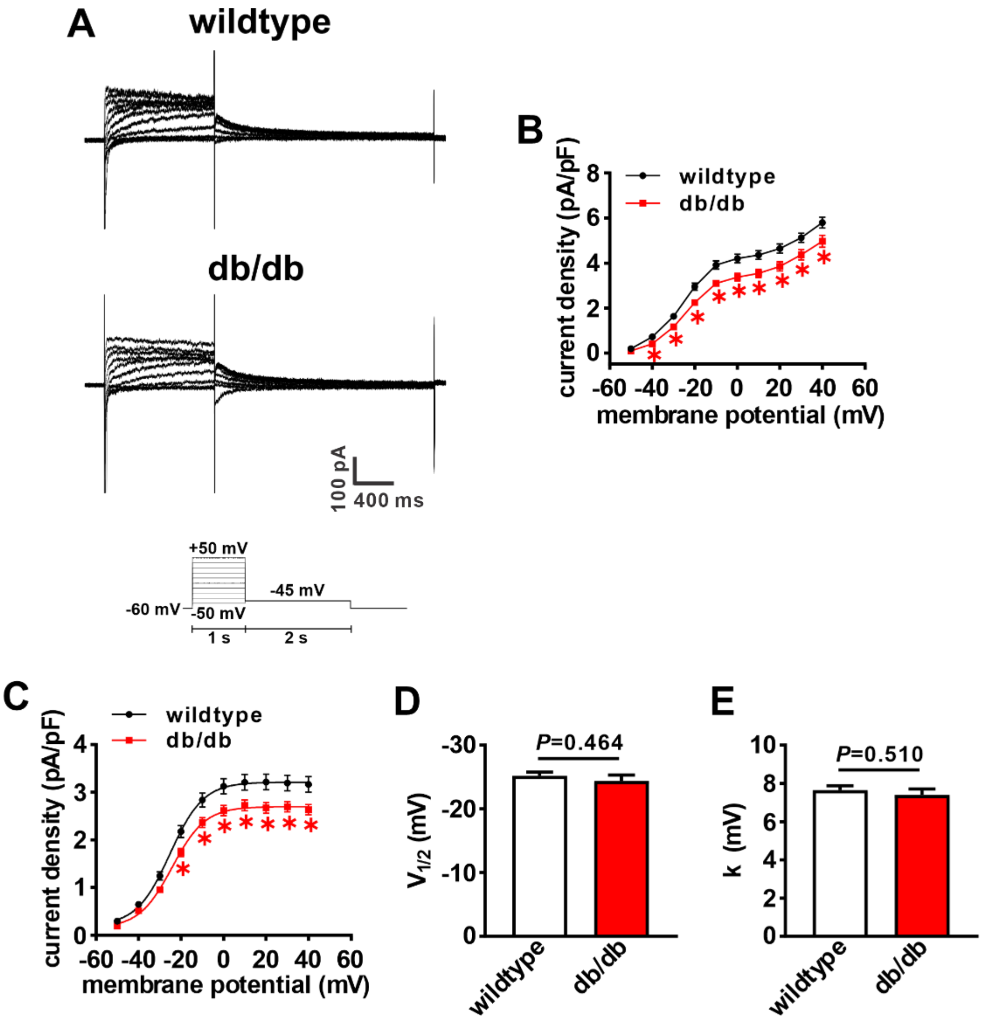

Figure S5: Rapidly activated delayed rectifier K^+ current (I_{Kr}) in db/db SAN myocytes. **A:** Representative I_K recordings in wildtype and db/db SAN myocytes. Voltage protocol shown at bottom of recordings. **B:** I_K IV relationship at the end of the 1 s voltage clamp step in wildtype (*n*=53) and db/db (*n*=42) SAN myocytes. **P*<0.05 vs wildtype by two-way repeated measures ANOVA with Holm-Sidak posthoc test. **C:** Boltzmann fit of I_{Kr} tail current in wildtype and db/db SAN myocytes. **P*<0.05 vs wildtype by two-way ANOVA with Holm-Sidak posthoc test. **D:** Voltage for 50% channel activation ($V_{1/2(act)}$) for I_{Kr} tail current in wildtype and db/db SAN myocytes. **E:** slope factor (k) for I_{Kr} tail currents in wildtype and db/db SAN myocytes. For panels **D** and **E** data analyzed by Student's *t*-test.

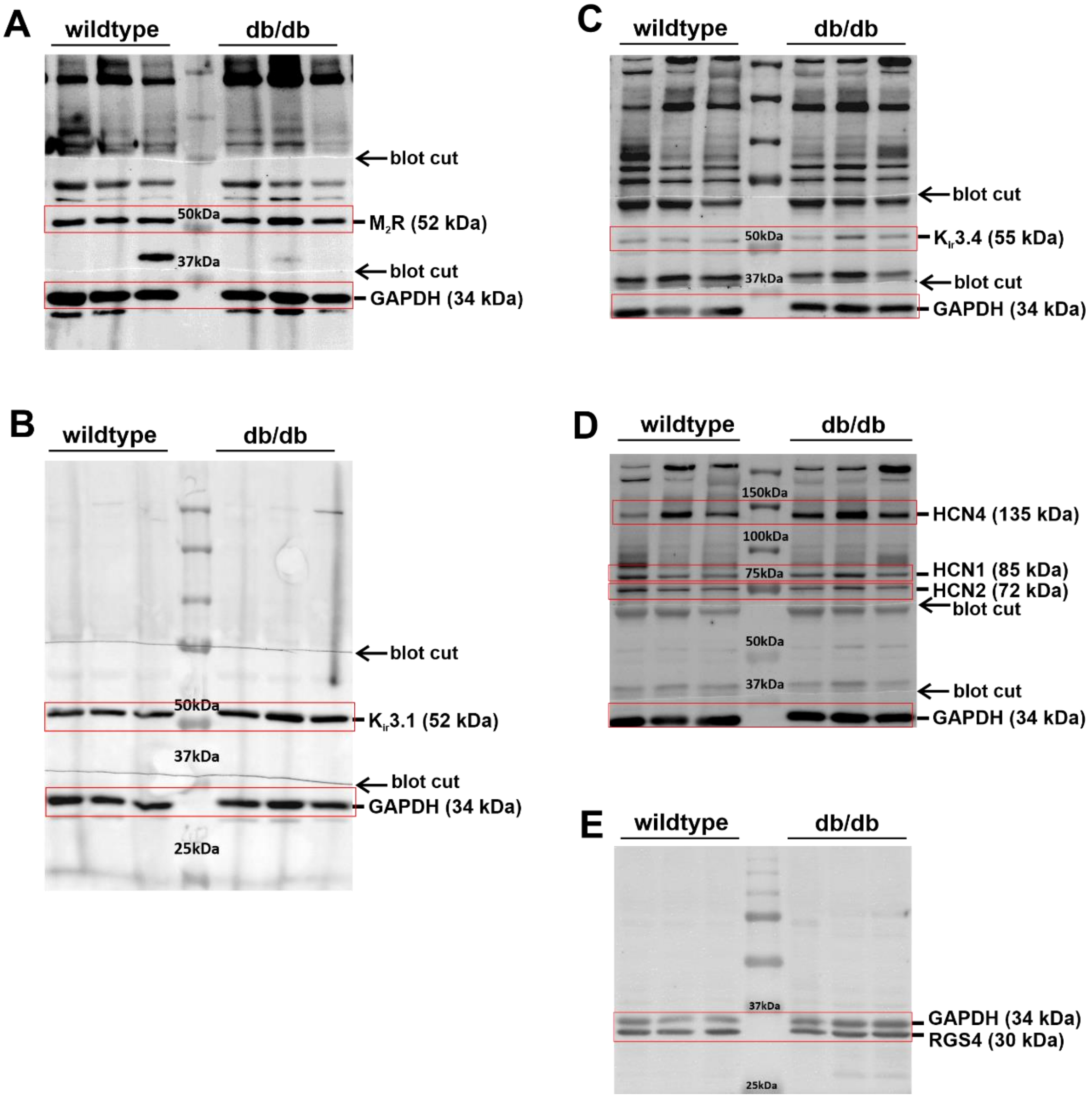

Figure S6: Uncropped Western blots. Full, uncropped representative Western blots for M_2R (**A**), $K_{ir}3.1$ (**B**), $K_{ir}3.4$ (**C**), HCN channels (**D**) and RGS4 (**E**) in the SAN in wildtype and db/db mice. HCN channels and $K_{ir}3.4$ were run on the same blot (panels C and D, which are presented at different contrasts). Red boxes indicate regions that were cropped and presented in figures within the manuscript. Note that membranes in panels A, B C and D were cut, as indicated by arrows, so that multiple proteins could be assessed on the same blot.

Supplemental Table 1: I_f activation kinetics in db/db mice

	**wildtype**		**db/db**	
	**baseline**	**CCh**	**baseline**	**CCh**
**$V_{1/2(act)}$ (mV)**	-107.9±0.4	-111.4±0.4*	-108.1±0.4	-112.3±.04*
**k (mV)**	13.4±0.4	13.0±0.4	14.3±0.3	14.3±0.4

$V_{1/2(act)}$, voltage for 50% channel activation; k, slope factor. *P<0.05 vs baseline by two-way repeated measures ANOVA with Holm-Sidak posthoc test; n=15 SAN myocytes for wildtype and 16 SAN myocytes for db/db.

Supplemental Table 2: Quantitative PCR primers

Gene of Interest	Forward Primer (5' → 3')	Reverse Primer (5' → 3')	Amplicon Length
Chrm2	AGTGTGGACAATTGGCTACTGG	ACCTTGTAGCGCCTATGTTCT	140
Kcnj3	AAACTCACTCTCATGTTCCG	TCCAGTTCAAGTTGGTCAAG	134
Kcnj5	AGATAGAAGGGCGAGGCAGA	CCCTTGGGGCTAACTTCTGG	192
HCN1	CTCTTTTTGCTAACGCCGAT	CATTGAAATTGTCCACCGAA	291
HCN2	CTTCACCAAGATCCTCAGTCTG	GGTCGTAGGTCATGTGGAAA	92
HCN4	CCAGGAGAAGTATAAACAGGTGGAGCG	GTTGATGATCTCCTCTCGAAGTGGCTC	169
gapdh	AATGGGGTGAGGCCGGTGCT	CACCCTTCAAGTGGGCCCCG	87

1. Hsueh, W. *et al.* Recipes for creating animal models of diabetic cardiovascular disease. *Circ. Res.* **100**, 1415-1427 (2007).
2. Coleman, D. L. Obese and diabetes: two mutant genes causing diabetes-obesity syndromes in mice. *Diabetologia* **14**, 141-148 (1978).
3. Bohne, L. J. *et al.* Electrical and structural remodeling contribute to atrial fibrillation in type 2 diabetic db/db mice. *Heart Rhythm* **18**, 118-129 (2021).
4. Egom, E. E. *et al.* Impaired sinoatrial node function and increased susceptibility to atrial fibrillation in mice lacking natriuretic peptide receptor C. *J. Physiol.* **593**, 1127-1146 (2015).
5. Jansen, H. J. *et al.* Atrial structure, function and arrhythmogenesis in aged and frail mice. *Sci. Rep.* **7**, 44336 (2017).
6. Mackasey, M. *et al.* Natriuretic Peptide Receptor-C Protects Against Angiotensin II-Mediated Sinoatrial Node Disease in Mice. *JACC Basic Transl. Sci.* **3**, 824-843 (2018).
7. Krishnaswamy, P. S. *et al.* Altered parasympathetic nervous system regulation of the sinoatrial node in Akita diabetic mice. *J. Mol. Cell. Cardiol.* **82**, 125-135 (2015).
8. Rose, R. A., Lomax, A. E., Kondo, C. S., Anand-Srivastava, M. B. & Giles, W. R. Effects of C-type natriuretic peptide on ionic currents in mouse sinoatrial node: a role for the NPR-C receptor. *Am. J. Physiol. Heart Circ Physiol* **286**, H1970-1977 (2004).
9. Hamill, O. P., Marty, A., Neher, E., Sakmann, B. & Sigworth, F. J. Improved patch-clamp techniques for high-resolution current recording from cells and cell-free membrane patches. *Pflugers Arch.* **391**, 85-100 (1981).
10. Lomax, A. E., Rose, R. A. & Giles, W. R. Electrophysiological evidence for a gradient of G protein-gated K+ current in adult mouse atria. *Br. J. Pharmacol.* **140**, 576-584 (2003).
11. Clark, R. B. *et al.* A rapidly activating delayed rectifier K+ current regulates pacemaker activity in adult mouse sinoatrial node cells. *Am. J. Physiol. Heart Circ Physiol* **286**, H1757-1766 (2004).
12. DiFrancesco, D. & Tromba, C. Inhibition of the hyperpolarization-activated current (if) induced by acetylcholine in rabbit sino-atrial node myocytes. *J. Physiol.* **405**, 477-491 (1988).
13. Mangoni, M. E. & Nargeot, J. Properties of the hyperpolarization-activated current (I(f)) in isolated mouse sino-atrial cells. *Cardiovasc. Res.* **52**, 51-64 (2001).
14. Springer, J. *et al.* The natriuretic peptides BNP and CNP increase heart rate and electrical conduction by stimulating ionic currents in the sinoatrial node and atrial myocardium following activation of guanylyl cyclase-linked natriuretic peptide receptors. *J. Mol. Cell. Cardiol.* **52**, 1122-1134 (2012).
15. Azer, J., Hua, R., Vella, K. & Rose, R. A. Natriuretic peptides regulate heart rate and sinoatrial node function by activating multiple natriuretic peptide receptors. *J. Mol. Cell. Cardiol.* **53**, 715-724 (2012).
16. Honjo, H., Boyett, M. R., Kodama, I. & Toyama, J. Correlation between electrical activity and the size of rabbit sino-atrial node cells. *J. Physiol.* **496 (Pt 3)**, 795-808 (1996).
17. Moghtadaei, M. *et al.* The impacts of age and frailty on heart rate and sinoatrial node function. *J. Physiol.* **594**, 7105-7126 (2016).
18. El Khoury, N. *et al.* Upregulation of the hyperpolarization-activated current increases pacemaker activity of the sinoatrial node and heart rate during pregnancy in mice. *Circulation* **127**, 2009-2020 (2013).
